# Multiplex Evaluation of Influenza Neutralizing Antibodies with Potential Applicability to In-Field Serological Studies

**DOI:** 10.1155/2014/457932

**Published:** 2014-07-03

**Authors:** Eleonora Molesti, Edward Wright, Calogero Terregino, Rafat Rahman, Giovanni Cattoli, Nigel J. Temperton

**Affiliations:** ^1^Viral Pseudotype Unit (Medway), School of Pharmacy, University of Kent, Central Avenue, Chatham Maritime, Kent ME4 4TB, UK; ^2^Viral Pseudotype Unit (Fitzrovia), Faculty of Science and Technology, University of Westminster, London W1W 6UW, UK; ^3^FAO, OIE, and National Reference Laboratory for Newcastle Disease and Avian Influenza, Istituto Zooprofilattico Sperimentale delle Venezie, Viale dell'Università 10, 35020 Legnaro, Italy

## Abstract

The increased number of outbreaks of H5 and H7 LPAI and HPAI viruses in poultry has major public and animal health implications. The continuous rapid evolution of these subtypes and the emergence of new variants influence the ability to undertake effective surveillance. Retroviral pseudotypes bearing influenza haemagglutinin (HA) and neuraminidase (NA) envelope glycoproteins represent a flexible platform for sensitive, readily standardized influenza serological assays. We describe a multiplex assay for the study of neutralizing antibodies that are directed against both influenza H5 and H7 HA. This assay permits the measurement of neutralizing antibody responses against two antigenically distinct HAs in the same serum/plasma sample thus increasing the amount and quality of serological data that can be acquired from valuable sera. Sera obtained from chickens vaccinated with a monovalent H5N2 vaccine, chickens vaccinated with a bivalent H7N1/H5N9 vaccine, or turkeys naturally infected with an H7N3 virus were evaluated in this assay and the results correlated strongly with data obtained by HI assay. We show that pseudotypes are highly stable under basic cold-chain storage conditions and following multiple rounds of freeze-thaw. We propose that this robust assay may have practical utility for in-field serosurveillance and vaccine studies in resource-limited regions worldwide.

## 1. Introduction

The increased number of outbreaks of H5 and H7 low pathogenicity avian influenza (LPAI) and high pathogenicity avian influenza (HPAI) viruses in poultry has major public and animal health implications and significant economic impact. The evaluation of evidence of influenza infection or vaccination efficiency in poultry species is assessed via the measurement of immunological responses against avian influenza viruses and serological assays representing an important tool for serosurveillance studies particularly in new outbreak locations. Although vaccination, combined with improved biosecurity has successfully prevented significant mortalities and production loss, on-going evolution of the virus requires the development of surveillance systems to ensure that vaccination continues to be effective [1].

Influenza A viruses infecting poultry are divided into two groups (HPAI and LPAI) on the basis of their ability to cause disease and with different human and animal health implications. Evidence [2, 3] supports the hypothesis that HPAI viruses arise as a result of mutations after the virus has been introduced from wild birds into poultry and thus, it is believed that LPAI viruses are the progenitors of the highly pathogenic variants [4]. It has been recognized as important to control not only HPAI viruses, but also LPAI strains in domestic poultry [5] despite current knowledge on the mechanisms of mutation from LPAI to HPAI being insufficient for predicting which influenza strains will mutate into HPAI variants [6, 7]. Despite significant efforts being put into the development of avian vaccines, serological surveillance represents one of the major tools for evaluating the immune state of avian populations especially for the ability of certain subtypes to mutate (antigenic drift mechanism) due to their long-term circulation among vaccinated populations [8]. Serology represents a powerful and sensitive approach for detecting the presence of avian influenza antibodies in a population but the occurrence of antigenic drift and shift must be taken into consideration as it can render subtype-specific serologic tests (HI or neutralization assays) less sensitive for new or emerging strains of influenza [9]. Additionally, serologic cross-reactivity with antigenically distinct influenza viruses can occur as a consequence of precedent vaccination or exposure, resulting in a more complicated interpretation of the serological findings. To address these issues, new assays are warranted as summarized within Appendix A of the Consultation Summary (May 2010) of an FAO-OIE-WHO Joint Technical consultation on Avian Influenza at the Human-Animal Interface (7–9 October, 2008, Verona, Italy) which makes the recommendation to “develop and validate more sensitive and specific tests for detecting antibodies to avian influenza viruses in avian and nonavian species including humans” [10]. As substitution rates are significantly higher in influenza HA and NA genes compared with internal genes, retroviral and lentiviral pseudotypes bearing HA and NA envelope glycoproteins devolved from the rest of the virus are ideal tools to monitor the effects of viral evolution on serological outcomes as previously shown [11–14]. They can be used as sensitive, low-containment assays for measuring antibody responses against HPAI and LPAI influenza strains [15] and potentially against all different influenza subtypes [16, 17] because, upon availability of the novel viral RNA/cDNA, HA/NA genes can be sequenced, readily cloned or custom synthesized, and pseudotyped lentiviral vectors prepared for use in neutralization assays. Therefore, this assay can be continually updated to measure the efficacy of current vaccines and therapeutics as well as serosurveillance. Also, the use of lentiviral pseudotypes has shown additional advantage compared to other serological assays since this system can potentially be adapted to a “multiplex format” with beneficial repercussions when large scale serological investigations need to be undertaken. In this study, the flexibility of the influenza pseudotype system has been exploited to develop a multiplex assay to study the neutralizing antibody responses directed against HAs belonging to Influenza Group 1 (HPAI H5N1 clade 1 A/Vietnam/1194/2004 and HPAI clade 2.1.3.2 A/Indonesia/5/2005) and Group 2 (HPAI H7N1 A/chicken/Italy/13474/1999). By the incorporation of different luciferase (Renilla and firefly) reporter genes into the lentiviral genome of two separate pseudotypes, each bearing an antigenically distinct envelope glycoprotein on its surface, the presence of neutralising antibodies against two influenza HAs has been evaluated within a single serum sample in a single assay plate well. Initially, sera from chickens vaccinated with a monovalent vaccine (H5N2) or from turkeys naturally infected during an H7N3 influenza outbreak were tested by pseudotype neutralization assay (pp-NT) assay using the “monoplex” format and serological results were compared to the standard reference HI test. Subsequently, H5 and H7 influenza pseudotypes were used for the screening of a panel of sera collected from chickens vaccinated with a bivalent vaccine (H5N9/H7N1) using a multiplex format where subtype-specific antibody responses in the same serum sample directed against H5 and H7 pseudotypes were evaluated exploiting the use of two different reporters and offering a new assay format for in-field serosurveillance and vaccine studies. We have also shown that these pseudotypes are highly stable at basic cold-chain storage conditions of −20°C and +4°C and after multiple rounds of freeze-thaw making these assays potentially applicable for use in-field in endemic areas as we have described recently for rabies and lyssaviruses [18, 19].

## 2. Materials and Methods

### 2.1. Serum Samples

All avian sera were provided by the FAO, OIE, and National Reference Laboratory for Newcastle Disease and Avian influenza (Istituto Zooprofilattico Sperimentale delle Venezie) and consisted of ten sera H5 positive collected from chickens vaccinated with the inactivated H5N2 (A/chicken/Hidalgo/28159-232/1994) vaccine (no. 1–10), ten sera H7 positive (no. 11–20) collected from turkeys during an Italian outbreak caused by an LPAI H7N3 virus (A/turkey/Italy/2002), ten sera positive for both H7 and H5 collected from chickens vaccinated with an inactivated bivalent vaccine produced with the LPAI H7N1 (A/chicken/1067/1999), and H5N9 (A/chicken/Italy/22A/1998) strains. Forty negative sera were included in the study and were obtained from chickens tested AI antibody-free by enzyme-linked immunosorbent assay (ELISA) and agarose gel immunodiffusion (AGID) assay using standard protocols described previously [20, 21]. Additionally, two hyperimmune sheep sera, SH454 raised against NIBRG-14 (H5N1 HA) and 02/294 raised against A/chicken/Italy/13474/1999 (H7N1 HA), were kindly provided by NIBSC.

### 2.2. Inhibition of Haemagglutination (HI) Test

All avian sera employed in the study were tested by HI at FAO, OIE, and National Reference Laboratory for Newcastle Disease and Avian influenza, Istituto Zooprofilattico Sperimentale delle Venezie with different reference antigens routinely used for avian influenza surveillance in Italy, namely, H5N2 (A/turkey/Italy/1980), H7N3 (A/turkey/Italy/9289/V02), H7N1 (A/Africa starling/England/983/1979), H5N9 (A/chicken/Italy/22A/1998), and H7N1 (A/chicken/Italy/1067/1999). For the HI tests, standard protocols were used as described previously [22].

### 2.3. Firefly Luciferase and Renilla Luciferase H5/H7 Pseudotypes

Lentiviral vector (carrying the luciferase reporter gene, pCSFLW) pseudotyped with HA envelope glycoproteins derived from the HPAI H5N1 viruses (clade 1 A/Vietnam/1194/2004 and clade 2.1.3.2 A/Indonesia/5/2005) and the HPAI H7N1 virus (A/chicken/Italy/13474/1999) were produced as described previously [23, 24], except that the neuraminidase activity was provided by a cognate NA plasmid in lieu of exogenous bacterial NA addition. In parallel, using the same transfection protocol and the same batch of HEK 293T/17 producer cells, HPAI H7 pseudotypes (A/chicken/Italy/13474/1999) carrying the Renilla luciferase gene (pCRLFW), were generated [24]. Using the firefly luciferase as marker for infection of HEK 293T/17 target cells, titration of H5, and H7 influenza pseudotype was carried out [23] in order to calculate the input virus dose required for the proceeding pseudotype-based neutralization assays. The titres of influenza pseudotypes were quantified by luminescence expression, expressed as relative luminescence units (RLU) measured by luminometer (GloMAX 96, Promega). Two controls were required for the titration: a negative control (cell only) and the Δ-envelope glycoprotein control (No HA and NA). In addition, for stability studies pCSLZW, expressing the lacZ gene [18], was used in conjunction with the clade 1 A/Vietnam/1194/2004 HA and exogenous bacterial NA (1 unit/mL; Sigma, UK) to produce lacZ pseudotype viruses, and infection of HEK 293T/17 cells was detected using the X-gal substrate as described by us previously [18]. Lentiviral pseudotypes bearing rabies CVS-11 [25] and HIV-1 [26] envelope glycoproteins were utilized for comparison.

### 2.4. Firefly Luciferase (Monoplex) pp-NT Assay

Serum samples (5 *μ*L) were twofold serially diluted in culture medium (DMEM GlutaMAX supplemented with 15% FBS and 1% Penicillin/Streptomycin) and mixed with pseudotype virus (500,000 RLU luciferase input) at a 1 : 1 v/v ratio. After incubation at 37°C for 1 hour, 1 × 10^4^ HEK 293T/17 cells were added to each well of a white 96-well flat-bottomed tissue culture plate. 48 hours later, pseudotype transduction titres obtained at each of a range of dilution points were expressed as RLU/mL, and an arithmetic mean was calculated. For each serum sample, RLUs were normalized and compared with the signal detected in the absence of pseudotype virus (equivalent to 100% neutralization) and the signal of the negative control (equivalent to 0% neutralization). The 50% inhibitory doses (IC_50_) were determined as the reciprocal of serum dilution resulting in a 50% reduction of a single round of infection (reporter gene mediated signal).

### 2.5. Firefly and Renilla (Multiplex) pp-NT Assay

To allow detection of neutralizing antibody responses against two different influenza viruses (H5 and H7) in the same well of a 96-well flat-bottomed tissue culture plate, fixed amounts (corresponding to 500,000 RLUs estimated by prior pseudotype titration) of both influenza pseudotypes (one containing the firefly reporter gene and the other the Renilla reporter gene) were added to each well in which twofold serially diluted serum samples (5 *μ*L) were dispensed together with cell culture medium (DMEM GlutaMAX supplemented with 15% FBS and 1% Penicillin/Streptomycin). After 48 hours, the neutralizing antibody responses against each subtype were detected by using the Dual-Glo reagent (Promega) which differentiates between the two reporter genes as detailed in the manufacturer's instructions, so that neutralizing antibody titre for each influenza pseudotype could be recorded for each serum sample concurrently.

### 2.6. Data Analysis and Sequence Analysis

Data analyses were undertaken using Excel and GraphPad Prism (Version 6). Antibody titres observed for H5 (A/Vietnam/1194/2004 and A/Indonesia/5/2005) and H7 (A/chicken/Italy/14374/1999) influenza pseudotypes when used in the monoplex and multiplex assays were expressed as geometric mean titer (GMT). Firstly, the IC_50_ values were calculated, as described above, and the serum dilution resulting in 50% neutralizing activity reduction for each serum sample (tested in duplicate) was transformed to logarithmic scale. Subsequently, the geometric mean of duplicate observations was calculated. Statistical analyses for all the data and correlation coefficients (Pearson's correlation analysis) were performed using GraphPad Prism. The radial tree and HA amino acid identity grid were constructed with MATLAB (MathWorks).

## 3. Results

### 3.1. Construction of H5 and H7 Lentiviral Pseudotypes

**Figure 1:**
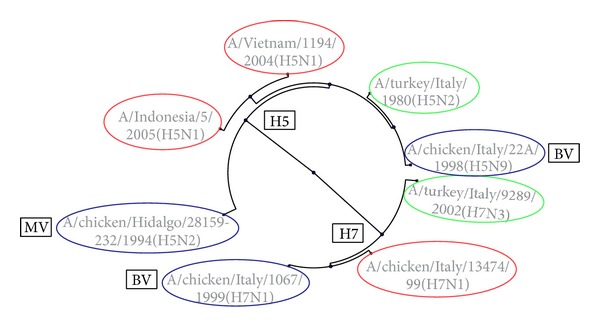
Radial phylogenetic tree showing the relationship between the full-length HA genes of vaccine and serological antigens (MATLAB Software). MV: monovalent vaccine strain, BV: bivalent vaccine strain. Viruses encircled in blue represent vaccine strains, in red represent pseudotype antigen strains, and in green represent HI antigen strains. The amino acid identity of these strains is shown in Figure 4.

We have constructed H5N1 and H7N1 pseudotypes (with A/Viet Nam/1194/2004 HA and NA, A/Indonesia/5/2005 HA and NA, and A/chicken/Italy/13474/1999 HA and NA) encoding the firefly luciferase reporter gene, and additionally, for use in a multiplex assay, an H7N1 pseudotype (with A/chicken/Italy/13474/1999 HA and NA) encoding a Renilla luciferase reporter. The phylogenetic relationship between these pseudotype serological antigens (and the other antigens utilized in this study) can be visualized on a radial tree in Figure 1. Using firefly luciferase as a marker for infection of HEK 293T/17 cells, it was shown that high titre functional pseudotypes bearing these three different envelope glycoprotein pairs were successfully produced (data not shown). Based on these virus titres, it was decided to use 500,000 RLU as the input virus dose for subsequent neutralization assays.

### 3.2. Stability of H5 Lentiviral Pseudotypes

**Figure 2:**
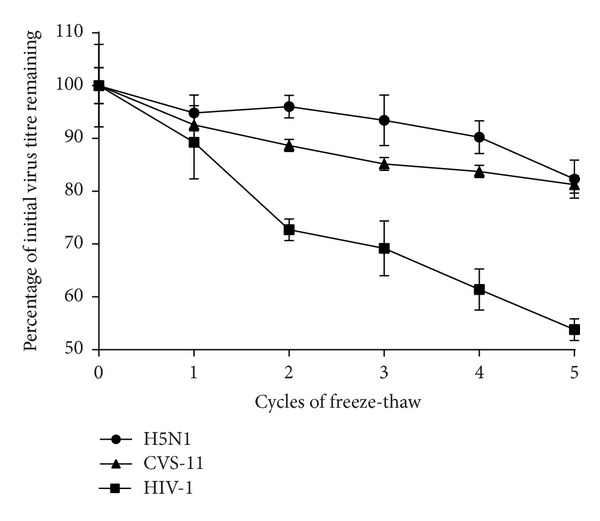
The influence of freeze-thaw cycles on pseudotype virus titre. The stability of H5N1 lacZ pseudotypes was evaluated by subjecting aliquots of virus to 5 cycles of freeze-thaw. Results for pseudotypes bearing the rabies virus (CVS-11) and HIV-1 envelope proteins are shown for comparison. One biological replicate of each pseudotype virus was used to generate three technical replicates. Error bars represent standard deviation.

**Figure 3:**
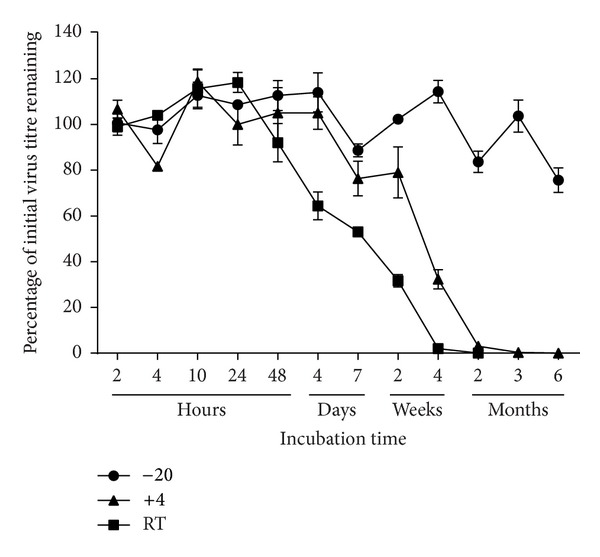
The influence of different storage temperatures on pseudotype virus titre. The stability of H5N1 lacZ pseudotypes was evaluated by storing aliquots of virus at different temperatures for up to 6 months. Pseudotype titres for the time course are given relative to the titre of virus stocks maintained at −80°C which is considered the optimal storage temperature for retroviral pseudotypes. One biological replicate pseudotype virus was used to generate three technical replicates. Error bars represent standard deviation.

The requirements and reliability of cold-chain storage in laboratories undertaking AI serology vary greatly, especially in resource limited regions of the world. Therefore, if these pseudotype-based assays are to be adopted in these regions in the future, it is assumed, primarily for cost reasons, that lacZ will be the reporter gene of choice and that these laboratories may have frequent disruptions to ideal pseudotype storage conditions (−80°C) or simply may have no access to a −80°C freezer. We therefore undertook a series of A/Viet Nam/1194/2004 HA pseudotype virus stability investigations by storage of this virus at the higher temperatures of −20°C (standard freezer), +4°C (standard fridge), and room temperature and by subjecting the pseudotype virus to multiple freeze-thaw cycles. The initial titre of the H5 lacZ pseudotype was 4.3 × 10^5^ IFU/mL and >80% infectivity remained after five cycles of freeze-thaw (Figure 2). In parallel, pseudotypes bearing rabies CVS-11 and HIV-1 envelope glycoproteins were subjected to the same freeze-thaw regimen and were found to lose approximately 4% and 9% activity, respectively, per freeze-thaw cycle (Figure 2). In relation to temperature storage variations and their effect on pseudotype viability, Figure 3 shows that H5 A/Viet Nam/1194/2004 HA pseudotypes stored at −20°C maintained infectivity (of >80% compared to storage at −80°C) for at least 6 months, making these assays readily applicable in the vast majority of laboratories worldwide. As also shown in Figure 3, these viruses could additionally be stored at +4°C for up to 4 weeks (with a 50% reduction in infectivity) and at room temperature (23°C) for 1 week (with a 50% reduction in infectivity).

### 3.3. Monoplex pp-NT Assay Using HPAI H5 and H7 Influenza Pseudotypes

Three panels of sera (H5 positive, H7 positive, and 40 negative serum samples) were initially tested using a monoplex pseudotype-based format. An initial pilot study was carried out where H5N1 hyperimmune (SH454) and H7N1 (02/294) sheep sera were tested for the ability to neutralize influenza pseudotypes bearing the HAs from H5N1 A/Vietnam/1194/2004 and H7N1 A/chicken/Italy/13474/1999. The H5 influenza pseudotypes were neutralized by the SH454 sera (100% inhibition of pseudotype entry at 1 : 1280 serum dilution) but not by the 02/294 sera whilst the H7 pseudotypes were neutralized by 02/294 (with a 100% inhibition at dilution 1 : 1280) but not by SH454. This lack of cross-neutralizing antibody response between H5 and H7 subtypes was consistent with the different clustering, within Group 1 and Group 2, of HA-subtypes based on phylogenetic relationship analysis of influenza subtypes. According to this analysis, H5 HA belongs to Group 1 “cluster 1” (together with H1, and H2, H6) and H7 HA belongs to Group 2 “cluster 7” (together with H10 and H15) [27]. Subsequently, a panel of ten sera collected from chickens vaccinated with a monovalent inactivated vaccine produced with an H5N2 strain (A/chicken/Hidalgo/232/1994) were run in order to more comprehensively evaluate the utility of this assay in an avian serological setting (tested using H5N1 A/Vietnam/1194/2004, H5N1 A/Indonesia/5/2005, and H7N1 A/chicken/13474/1999 pseudotyped particles). Serological profiles obtained by pp-NT assays were also compared with those obtained by HI tests. All ten sera positive by HI test using an H5N2 A/chicken/Italy/1080 reference antigen (with titres ranging from 1 : 128 to 1 : 2048) were also confirmed positive by pp-NT assay using H5N1 A/Vietnam/1194/2004 (GMT range 1 : 113 to 1 : 2560) and H5N1 A/Indonesia/5/2005 (GMT range 1 : 60 to 1 : 2560) (Table 1). When this panel was tested against H7 HPAI pseudotypes, six of ten sera were also found positive with GMT ranging from 1 : 28 to 1 : 113 (Table 1).

**Figure 4:**
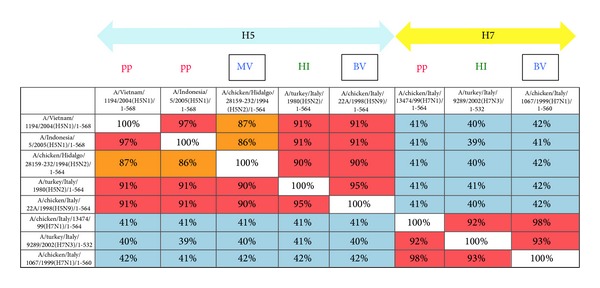
Amino acid identity grid for pseudotype, HI, and vaccine antigen strains. MV: monovalent vaccine antigen, BV: bivalent vaccine antigen, pp: pseudotype antigen, and HI: haemagglutination inhibition assay antigen.

**Figure 5:**
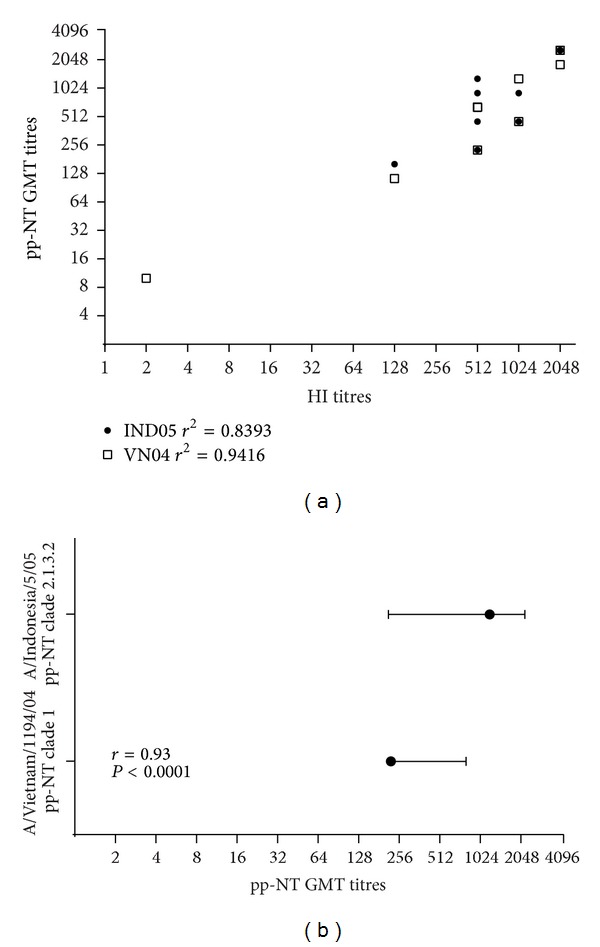
(a) Comparison of pp-NT with HI titers. Scatterplots showing the correlation of antibody logarithmic titers measured by pp-NT versus HI. For the pp-NT assay, HPAI H5 from A/Vietnam/1194/04 VN04 and A/Indonesia/5/05 (IND05) were tested. Pearson's correlation analysis was carried out and the coefficient of determination (*r* ^2^) for each strain reported on the graph. (b) Paired *t*-test performed by using GraphPad.

As shown in Figure 5(a), titres obtained via HI correlated strongly with titres obtained using HPAI H5 pseudotypes belonging to two different clades: clade 1 A/Vietnam/1194/2004 (*r* = 0.87, *P* < 0.0001) and clade 2.1.3.2 A/Indonesia/5/2005 (*r* = 0.87, *P* < 0.0002) despite the fact the HAs used in the two serological assays were not optimally matched. The percentage amino acid identities between the pseudotype antigens, the HI antigen, and the vaccine antigen are shown in Figure 4.

It was also observed (Figure 5(b)) that all ten sera neutralized both H5 HPAI pseudotyped viruses but with a different magnitude; it was found that the absolute titers (expressed as mean ± SD) of H5 A/Vietnam/1194/2004 (222.96) were significantly lower than those obtained for the same panel tested by A/Indonesia/5/2005 (1206.2) as confirmed by *P* value <0.0001 and *r* = 0.93 when analyzed using Student's *t*-Test (paired data set) (Figure 5).

Next, a panel of ten sera obtained from turkeys naturally infected with an H7N3 strain with titres ranging from low (1 : 32) to high (1 : 128) as tested by HI (HI reference antigen: H7N3 A/turkey/Italy/9289/V02) were subsequently tested using H7 pseudotypes A/chicken/Italy/13474/1999 and H5 A/Vietnam/1194/2004. All ten sera (no. 11 to no. 20) were found positive when tested against H7 pseudotypes with GMT range from 1 : 28 to 1 > 2560 while only one serum sample was positive (GMT of 1 : 28) against H5 A/Vietnam/1194/2004 (Table 2).

**Figure 6:**
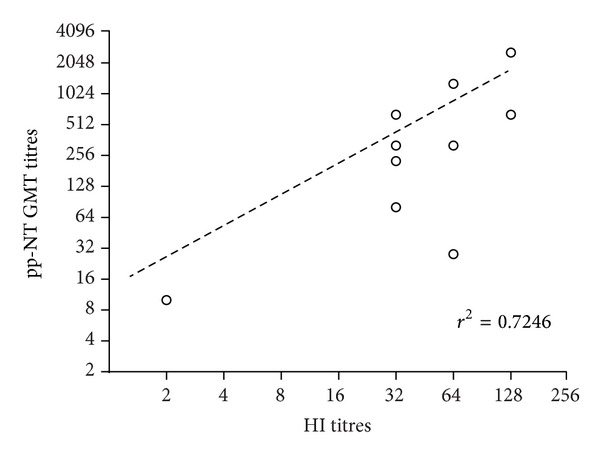
Comparison of pp-NT with HI titers. Scatterplots showing the correlation of antibody logarithmic titers measured by pp-NT (using HPAI H7 A/chicken/Italy/13474/99 pp) versus HI (HI antigen: H7N3 A/ty/Italy/9289/V02). The total number of sera was 51. Graph shows the linear regression fitted to the data using GraphPad.

As previously shown for the panel of H5 positive sera, a comparative serological approach was undertaken in order to assess whether the results obtained with the pseudotype neutralization assay reflected those obtained with HI test using a regression analysis on paired datasets (generated from all 51 samples comprising also the negative sera) and the Pearson's correlation test. The results of this analysis revealed a highly statistically significant correlation (*P* < 0.001) between antibody titers obtained with both assays. The correlation coefficient between pp-NT and HI for the panel of H7 positive was 0.72 (Figure 6).

### 3.4. Multiplex Assay by Using HPAI H5 and H7 Influenza Pseudotypes Expressing Firefly and Renilla Luciferase Reporter Gene

For a panel of sera collected from chickens vaccinated with an inactivated bivalent vaccine (BV) produced with the avian influenza vaccine strains H7N1 (A/chicken/Italy/1067/1999, LPAI) and H5N9 (A/chicken/Italy/22A/1998, LPAI), monoplex (as described before) and multiplex (by using firefly/Renilla) pp-NT assays were undertaken against H5 A/Vietnam/1194/2004 and H7 A/chicken/Italy/13474/1999 pseudotypes. As shown in Table 3, 9/10 sera were confirmed positive when tested by the monoplex assay against H5 A/Vietnam/1194/2004 with titres (expressed as GMT) ranging between 1 : 28 to 1 : 905; only one serum sample (n. H5+7s3), positive by HI (1 : 64), was found negative by pp-NT (GMT = 10). Neutralizing antibody responses against H5 A/Indonesia/5/2005 were also found positive (10/10 sera) with GMTs ranging between 1 : 28 and 1 : 905 and showing, as seen for the panel of H5 positive sera (Table 1), overall higher GMTs compared to H5N1 pseudotypes belonging to clade 1. Also for H7N1 pseudotypes the GMTs mirrored those obtained with H5N1 pseudotypes (GMTs from 1 : 40 to 1 : 453) with one serum sample (n. H5+7s3) negative (GMT = 10) and two sera at the proposed positive threshold of 1 : 40.

**Figure 7:**
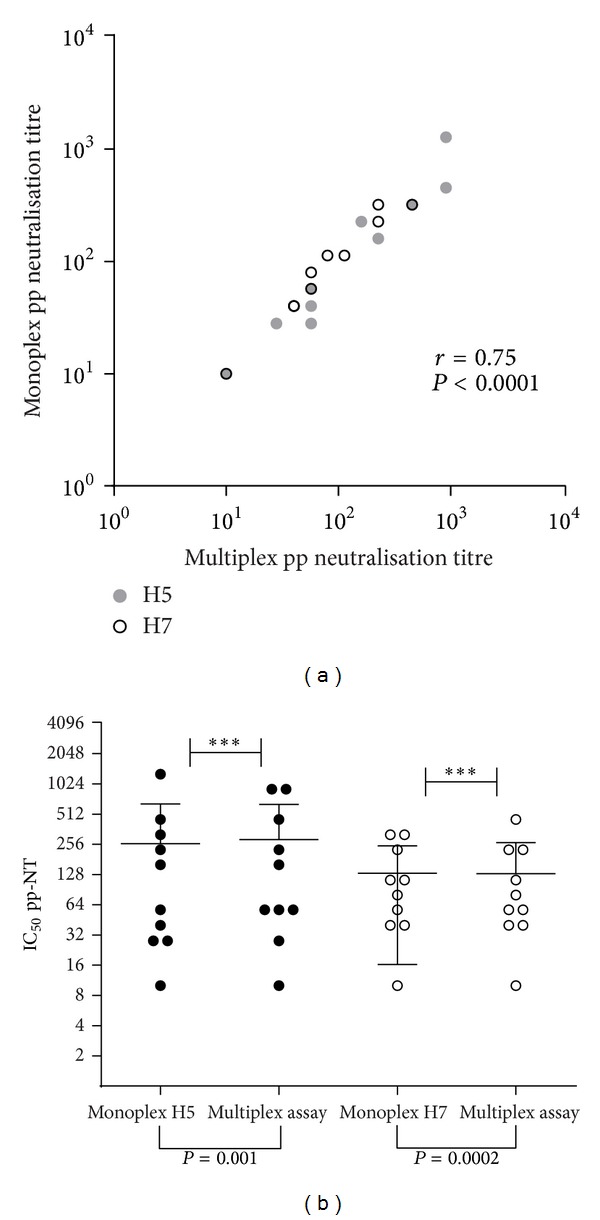
(a) Correlation of monoplex versus multiplex IC_50_ pseudotype neutralization titres for sera collected from chickens vaccinated with an inactivated bivalent vaccine produced with the AI strains H7N1 (A/ck/Italy/1067/99, LPAI) and H5N9 (A/ck/Italy/22A/98, LPAI). Antibody titres between monoplex and multiplex assays correlate when tested with both H5 and H7 pseudotypes. Neutralizing titres against H5 A/Vietnam/1194/2004 (grey dots) and H7 A/chicken/Italy/13474/1999 (empty circles) were determined in separate wells (single) or in the same well (multiplex). Correlation coefficient and *P* values were calculated using Pearson's correlation. Plot drawn with GraphPad. (b) IC_50_ values for each sera tested by monoplex and multiplex pp-NT assays using H5 A/Vietnam/1194/04 (firefly luciferase gene) and H7 A/chicken/Itlay/13474/1999 (carrying firefly luciferase and Renilla luciferase gene) were calculated and plotted (the wide horizontal bar represents the means of IC_50_ titres). Results were subsequently analyzed by performing Student's *t*-test on the paired dataset.

Subsequently, neutralizing antibody titres, obtained when standard and dual H5/H7 assays were undertaken (GMT reported in Table 3), revealed a strong correlation between the results of the standard and dual pp-NT assays (0.86; *P* < 0.0001; Pearson's correlation; Figure 7).

Neutralizing antibody titres obtained by monoplex assay (using H5 A/Vietnam/1194/2004 carrying the firefly luciferase gene) mirrored those obtained when the same panel of sera were tested against H5 A/Vietnam/1194/2004 (firefly gene) mixed with H7 A/chicken/13474/1999 carrying the Renilla gene (*r* = 0.85, *P* = 0.001). Similar results were observed for antibody responses against H7 A/chicken/13474/1999 tested in monoplex and multiplex assays (*r* = 0.91, *P* = 0.0002) (Figure 7). The magnitude of neutralizing antibody responses observed by pp-NT assays reflected those obtained by the standard HI test although the HI test had to be performed for evaluating HA-mediated antibody responses versus both influenza antigens H5N9 (A/chicken/Italy/22A/1998) and H7N1 (A/chicken/Italy/1067/1999) (Table 3).

## 4. Discussion and Conclusion

Since 1997, H5 and H7 outbreaks in domestic poultry have been increasing in frequency and it is likely to be due to a complex set of factors such as improved diagnostic tools, climate fluctuations, and changes in trade flows of poultry products [28]. One logical step to understand and limit the possible spread of avian influenza viruses to humans and to control the circulation amongst avian species is the monitoring of AI virus exposure in poultry initially via identification of active infections. However, due to the ability of influenza viruses to circumvent immunity acquired through infection or vaccination by progressive antigenic drift, serological surveillance of avian samples is also particularly important [29, 30]. Serological techniques play a key role in various aspects of influenza surveillance, vaccine development, and evaluation and they can be used to assess the presence of antibodies to past infections and responses to a circulating influenza strain or vaccine components [31]. From a veterinary point of view, serological and virological surveillances are necessary not only as monitoring systems for AI viruses circulating among poultry species but also as a prevention and control tool for those strains with possible pandemic potential [32, 33].

Recent studies have provided the impetus that the future of avian serology, rather than moving towards a single assay approach, is the implementation of a strategy that involves conventional and novel technologies to be used in conjunction with validated and standard tests. Comparative serology aims to achieve a more holistic view of the serological response and newer assays like the pseudotype-based neutralization assay presented in this study are key [14, 23]. We have shown previously that retroviral pseudotypes (MLV) based on A/Viet Nam/1194/2004 can be used to measure antibody responses in chickens immunized with H5N1, H5N2, H5N3, H5N7, and H5N9 avian viruses [11]. In this current study, lentiviral pseudotypes have been employed to form the basis for the development of a multiplex reporter (firefly luciferase and Renilla luciferase) neutralization assay for H5 and H7 subtype viruses. This pseudotype system allows the measurement of neutralizing antibody responses against two antigenically distinct AI HA envelope glycoproteins in the same avian serum sample. The individual components employed for the construction of the pseudotypes used for this multiplex assay have been chosen from a set of interchangeable plasmids which we have available for assay development. These are retroviral and lentiviral plasmids coding for the* gag*-*pol* core structural proteins, HA and NA expression plasmids, and retroviral vectors incorporating the reporter gene. Firefly and Renilla luciferases were employed in this study, but potentially a wide range of reporter genes can be used in these assays (green fluorescence protein (GFP), red (RFP)/yellow (YFP), secreted embryonic alkaline phosphatase (SEAP), and lac-Z) [18, 19, 34, 35]. In order for pseudotype assays to have wide applicability and deployment potential within different laboratories worldwide, the availability of different reporter systems is highly desirable. The HIV-based GFP reporter plasmid (pCSGW), which we have described previously in the context of pseudotype-based neutralization assays [23], has been modified by PCR subcloning to express alternative reporter genes. These are firefly luciferase (pCSFLW), Renilla luciferase (pCSRLW), and* lac*-Z (pCSLZW) [19, 24]. Of the three types, the luciferase reporter based assays are the most sensitive and reproducible and also the simplest to use in terms of hands-on time and downstream data analysis. This was the reason they were chosen for the serological assays described in this current study. However, due to the relatively high cost of the necessary reagents (luciferase assay) and necessity for specialized equipment (luminometer), luciferase assays may have limited applicability for laboratories in resource poor regions. GFP based assays do not require any supplementary reagents but do necessitate specialized equipment (fluorescent microscope or 96-well plate flow cytometry facility). The cost of a basic fluorescent microscope however is now under $5000 making this technology applicable for middle to low-resource lab deployment. The lac-Z based assays are the most cost effective as the necessary reagents are cheaply available and specialized equipment is unnecessary making them ideal for deployment in resource poor areas where serosurveillance in domestic poultry is likely to be carried out. In addition to firefly/Renilla (used in this study), it is technically feasible to multiplex pairs of pseudotype viruses carrying GFP/RFP and lacZ/SEAP combinations for low-resource laboratory use. Lac-Z was thus chosen as the reporter gene of choice for the “in-field” applicability studies involving the freeze-thawing and storing of pseudotype viruses outside of a −80°C facility as was used recently for similar studies with lyssavirus pseudotypes [18]. Our results showed them to be highly suitable for such use as they were stable over time at different storage temperatures and when subjected to multiple cycles of freeze-thaw. Interestingly the pseudotype virus bearing the HIV envelope glycoprotein was significantly more sensitive to the freeze-thaw procedure than the viruses bearing influenza or rabies virus glycoproteins. This is most likely due to the fact that HIV glycoprotein is relatively unstable when frozen. Additionally, with the multiplex assay using the dual reporter gene system, the interassay variability is likely to be reduced since only a single serum dilution series needs to be performed. The same preparation of target cells is used for two viruses and the antibody response to the H5 subtype virus may serve as an internal “serocontrol” for the antibody response to the H7 subtypes and vice versa as two separate luciferase reporters were employed.

Results collected from pp-NT assays were statistically significant when performed in monoplex and multiplex from both H5 (*P* = 0.001) and H7 (*P* = 0.0002) influenza strains with results from the monoplex mirroring those obtained with the multiplex assay (Figure 7). This system could be subsequently refined with the possibility of increasing the multiplexing capability (use of more reporter systems by detecting luminescence and fluorescence signals, e.g., GFP/RFP with firefly/Renilla luciferase) and it could readily be adapted to high-throughput if large serum panels are used. There are also beneficial economic implications to the use of this assay since the antibody responses against two viruses do not require high-containment facilities and relatively fewer reagents than HI and MN tests. The pp-NT assay described here is both “serum sparing” and “antigen sparing” as only ≤5 *μ*L and, especially for certain HPAI strains, less than 10 *μ*L (corresponding to a pseudotype input of 10e6 RLU) pseudotypes per 96-well plate is required. It is possible with the multiplex pp-NT assay to measure neutralizing antibody responses against large panels of H5/H7 influenza viruses and drift variants faster and more accurately than laborious wild-type virus microneutralization, thus providing comprehensive data on antigenic evolution of avian influenza viruses.

Moreover, the major limitations to the use of HI assay are that it is not practical for general influenza A screening with significant level of intralaboratory variability as demonstrated in human serology [36]. It requires a greater amount of sera and the occurrence of cross-reactivity between subtypes needs to be taken into account. On the contrary, pseudotype particles have been shown to be particularly sensitive with the potential to detect antibody responses and variations within influenza subclades and also showing statically significant correlation when compared to the HI test (Table 1) (Figures 5 and 6) [23]. Recent studies have raised the possibility that the lower incorporation of HA spikes into lentiviral pseudotypes, compared to the wild virus, makes pseudotypes more sensitive by allowing the binding of antibodies to the antigenic sites on the HA head and also on the HA stalk [37, 38]. Based on the data obtained in this study, future refinement of this assay is warranted and it contributes towards the recommendations for the development of new assays as outlined in the FAO-OIE-WHO Joint Technical Consultation document [10]. In addition, this study provides the basis for future composite studies where collaborating laboratories can be involved to determine whether the level of intra- and interlaboratory variability in pp-NT assay is lower than that found with HI or indeed MN enabling the pp-NT assay to become accepted for large scale testing, not only in the context of avian and human influenza surveillance but also for integrated surveillance of other “neglected” influenza strains (circulating in horses, pigs, seals, and dogs for e.g.) [39].

## Figures and Tables

**Table 1:** Comparison of neutralizing activity of a panel of sera collected from chickens vaccinated with an inactivated H5N2 monovalent vaccine.

Serum number	H5 positive sera (H5N2 monovalent vaccine)			
	GMT titres H5N1 A/Vietnam/1194/04	GMT titres H5N1 A/Indonesia/5/05	GMT titres H7N1 A/chicken/13474/99	HI titres H5N2 A/chicken/Italy/80
1	2560	2560	28	2048
2	2560	2560	113	2048
3	640	1280	10	512
4	1810	2560	10	2048
5	453	453	10	1024
6	1280	905	28	1024
7	226	226	10	512
8	113	160	113	128
9	640	905	28	512
10	640	453	57	512
H5N1+	1280	—	—	—
Negatives (40 tot.)	≤1 : 10	≤1 : 10	—	1 : 2

GMT titres were calculated and expressed as the reciprocal of serum dilution at which a 50% inhibition of pseudotype (IC_50_) entry was observed.

**Table 2:** Comparison of neutralizing activity of panel of sera collected from naturally infected turkeys with H7N3 A/turkey/Italy/2002.

Serum number	H7 positive sera (H7N3 positive, naturally infected)		
	GMT titres H7N1 A/chicken/13474/99	GMT titres H5N1 A/Vietnam/1194/04	HI titres A/ty/Italy/9289/V02 H7N3
11	320	10	64
12	320	10	32
13	2560	10	128
14	28	10	64
15	2560	10	128
16	80	10	32
17	226	10	32
18	640	28	32
19	640	10	128
20	1280	10	64
H7N1+	1 : 1280	≤1 : 10	—
Negatives (40 tot.)	≤1 : 10	—	1 : 2

GMT titres were calculated and expressed as the reciprocal of the serum dilution at which 50% inhibition of pseudotype (IC_50_) entry was observed.

**Table 3:** Evaluation of antibody responses in sera collected from chickens vaccinated with a bivalent vaccine (H5/H7) by using the monoplex and multiplex assay formats.

Serum number	HI H7N1	H7 pp-NT monoplex A/ck/Italy/13474/99	H7 pp-NT multiplex (with H5 VN04)	Serum number	HI H5N9	H5 pp-NT monoplex VN04	H5 pp-NT monoplex H5 IND05	H5 pp-NT multiplex (with H7)
H5+7s1	32	40	40	H5+7s1	32	57	80	40
H5+7s2	16	57	80	H5+7s2	32	57	80	28
H5+7s3	32	10	10	H5+7s3	64	10	28	10
H5+7s4	16	40	40	H5+7s4	16	28	40	28
H5+7s5	32	453	320	H5+7s5	64	905	453	453
H5+7s6	32	226	226	H5+7s6	32	160	160	226
H5+7s7	16	113	113	H5+7s7	32	226	320	160
H5+7s8	16	57	57	H5+7s8	64	57	80	57
H5+7s9	16	80	113	H5+7s9	32	453	905	320
H5+7s10	32	226	320	H5+7s10	32	905	905	1280

Values are reported as geometric mean titres. Left side: values for H7 A/chicken/Italy/13474/99 tested in monoplex and multiplex are reported. Right side: values for H5 A/Vietnam/1194/04 tested in monoplex and multiplex are reported and the panel of sera was additionally tested against A/Indonesia/5/05.

## References

[1] Capua I, Marangon S (2007). Control and prevention of avian influenza in an evolving scenario. *Vaccine*.

[2] Röhm C, Horimoto T, Kawaoka Y, Süss J, Webster RG (1995). Do hemagglutinin genes of highly pathogenic avian influenza viruses constitute unique phylogenetic lineages?. *Virology*.

[3] Banks J, Speidel ES, Moore E (2001). Changes in the haemagglutinin and the neuraminidase genes prior to the emergence of highly pathogenic H7N1 avian influenza viruses in Italy. *Archives of Virology*.

[4] García M, Crawford JM, Latimer JW, Rivera-Cruz E, Perdue ML (1996). Heterogeneity in the haemagglutinin gene and emergence of the highly pathogenic phenotype among recent H5N2 avian influenza viruses from Mexico. *Journal of General Virology*.

[5] Capua I, Marangon S, Dalla Pozza M, Santucci U (2000). Vaccination for avian influenza in Italy. *Veterinary Record*.

[6] Comin A, Klinkenberg D, Marangon S, Toffan A, Stegeman A (2011). Transmission dynamics of low pathogenicity avian influenza infections in Turkey flocks. *PLoS ONE*.

[7] Sharp GB, Kawaoka Y, Wright SM, Turner B, Hinshaw V, Webster RG (1993). Wild ducks are the reservoir for only a limited number of influenza A subtypes. *Epidemiology and Infection*.

[8] Lee C-W, Senne DA, Suarez DL (2004). Effect of vaccine use in the evolution of Mexican lineage H5N2 avian influenza virus. *Journal of Virology*.

[9] Wibawa H, Henning J, Waluyati DE (2012). Comparison of serological assays for detecting antibodies in ducks exposed to H5 subtype avian influenza virus. *BMC Veterinary Research*.

[10] Anderson T, Capua I, Dauphin G (2010). FAO-OIE-WHO Joint Technical Consultation on avian Influenza at the human-animal interface. *Influenza and Other Respiratory Viruses*.

[11] Temperton NJ, Hoschler K, Major D (2007). A sensitive retroviral pseudotype assay for influenza H5N1-neutralizing antibodies. *Influenza and Other Respiratory Viruses*.

[12] Garcia J-M, Lagarde N, Ma ESK, de Jong MD, Peiris JSM (2010). Optimization and evaluation of an influenza A (H5) pseudotyped lentiviral particle-based serological assay. *Journal of Clinical Virology*.

[13] Nefkens I, Garcia J-M, Ling CS (2007). Hemagglutinin pseudotyped lentiviral particles: characterization of a new method for avian H5N1 influenza sero-diagnosis. *Journal of Clinical Virology*.

[14] Desvaux S, Garcia JM, Nguyen TD (2012). Evaluation of serological tests for H5N1 avian influenza on field samples from domestic poultry populations in Vietnam: consequences for surveillance. *Veterinary Microbiology*.

[15] Molesti E, Cattoli G, Ferrara F, Böttcher-Friebertshäuser E, Terregino C, Temperton N (2013). The production and development of H7 Influenza virus pseudotypes for the study of humoral responses against avian viruses. *Journal of Molecular and Genetic Medicine*.

[16] Corti D, Voss J, Gamblin SJ (2011). A neutralizing antibody selected from plasma cells that binds to group 1 and group 2 influenza A hemagglutinins. *Science*.

[17] Ferrara F, Molesti E, Böttcher-Friebertshäuser E (2012). The human transmembrane protease serine 2 is necessary for the production of group 2 influenza A virus pseudotypes. *Journal of Molecular and Genetic Medicine*.

[18] Wright E, McNabb S, Goddard T (2009). A robust lentiviral pseudotype neutralisation assay for in-field serosurveillance of rabies and lyssaviruses in Africa. *Vaccine*.

[19] Wright E, Hayman DTS, Vaughan A (2010). Virus neutralising activity of African fruit bat (*Eidolon helvum*) sera against emerging lyssaviruses. *Virology*.

[20] OIE (2013). *Manual of Diagnostic Tests and Vaccines for Terrestrial Animals: Avian Influenza*.

[21] Cattoli G, Milani A, Temperton N (2011). Antigenic drift in H5N1 avian influenza virus in poultry is driven by mutations in major antigenic sites of the hemagglutinin molecule analogous to those for human influenza virus. *Journal of Virology*.

[22] Terregino C, Toffan A, Cilloni F (2010). Evaluation of the protection induced by avian influenza vaccines containing a 1994 Mexican H5N2 LPAI seed strain against a 2008 Egyptian H5N1 HPAI virus belonging to clade 2.2.1 by means of serological and in vivo tests. *Avian Pathology*.

[23] Molesti E, Milani A, Terregino C, Cattoli G, Temperton NJ (2013). Comparative serological assays for the study of H5 and H7 avian influenza viruses. *Influenza Research and Treatment*.

[24] Alberini I, Del Tordello E, Fasolo A (2009). Pseudoparticle neutralization is a reliable assay to measure immunity and cross-reactivity to H5N1 influenza viruses. *Vaccine*.

[25] Wright E, Temperton NJ, Marston DA, McElhinney LM, Fooks AR, Weiss RA (2008). Investigating antibody neutralization of lyssaviruses using lentiviral pseudotypes: a cross-species comparison. *Journal of General Virology*.

[26] Helseth E, Kowalski M, Gabuzda D, Olshevsky U, Haseltine W, Sodroski J (1990). Rapid complementation assays measuring replicative potential of human immunodeficiency virus type 1 envelope glycoprotein mutants. *Journal of Virology*.

[27] Sui J, Hwang WC, Perez S (2009). Structural and functional bases for broad-spectrum neutralization of avian and human influenza A viruses. *Nature Structural and Molecular Biology*.

[28] Capua I, Alexander DJ (2004). Avian influenza: recent developments. *Avian Pathology*.

[29] Sullivan HJ, Blitvich BJ, VanDalen K, Bentler KT, Franklin AB, Root JJ (2009). Evaluation of an epitope-blocking enzyme-linked immunosorbent assay for the detection of antibodies to influenza A virus in domestic and wild avian and mammalian species. *Journal of Virological Methods*.

[30] Capua I, Marangon S (2006). Control of avian influenza in poultry. *Emerging Infectious Diseases*.

[31] Katz JM, Hancock K, Xu X (2011). Serologic assays for influenza surveillance, diagnosis and vaccine evaluation. *Expert Review of Anti-Infective Therapy*.

[32] Naeem K, Naurin M, Rashid S, Bano S (2003). Seroprevalence of avian influenza virus and its relationship with increased mortality and decreased egg production. *Avian Pathology*.

[33] Cattoli G, Terregino C (2008). New perspectives in avian influenza diagnosis. *Zoonoses and Public Health*.

[34] Rizzo MA, Davidson MW, Piston DW (2009). Fluorescent protein tracking and detection: fluorescent protein structure and color variants. *Cold Spring Harbor Protocols*.

[35] Temperton NJ, Chan PK, Simmons G (2005). Longitudinally profiling neutralizing antibody response to SARS coronavirus with pseudotypes. *Emerging Infectious Diseases*.

[36] Stephenson I, Heath A, Major D (2009). Reproducibility of serologic assays for influenza virus A (H5N1). *Emerging Infectious Diseases*.

[37] Pica N, Hai R, Krammer F (2012). Hemagglutinin stalk antibodies elicited by the 2009 pandemic influenza virus as a mechanism for the extinction of seasonal H1N1 viruses. *Proceedings of the National Academy of Sciences of the United States of America*.

[38] Corti D, Suguitan AL, Pinna D (2010). Heterosubtypic neutralizing antibodies are produced by individuals immunized with a seasonal influenza vaccine. *The Journal of Clinical Investigation*.

[39] Capua I, Alexander D (2010). Perspectives on the global threat: the challenge of avian influenza viruses for the world’s veterinary community. *Avian Diseases*.

